# Cortical and Subcortical Neural Correlates for Respiratory Sensation in Response to Transient Inspiratory Occlusions in Humans

**DOI:** 10.3389/fphys.2018.01804

**Published:** 2018-12-18

**Authors:** Pei-Ying S. Chan, Chia-Hsiung Cheng, Yu-Ting Wu, Changwei W. Wu, Ho-Ling A. Liu, Fu-Zen Shaw, Chia-Yih Liu, Paul W. Davenport

**Affiliations:** ^1^Department of Occupational Therapy and Healthy Aging Center, Chang Gung University, Taoyuan, Taiwan; ^2^Department of Psychiatry, Chang Gung Memorial Hospital at Linkou, Taoyuan, Taiwan; ^3^Laboratory of Brain Imaging and Neural Dynamics (BIND Lab), Chang Gung University, Taoyuan, Taiwan; ^4^Graduate Institute of Mind, Brain and Consciousness, Taipei Medical University, Taipei, Taiwan; ^5^Brain and Consciousness Research Center, Taipei Medical University-Shuang Ho Hospital, New Taipei, Taiwan; ^6^Department of Imaging Physics, The University of Texas MD Anderson Cancer Center, Houston, TX, United States; ^7^Department of Psychology, National Cheng-Kung University, Tainan, Taiwan; ^8^Department of Physiological Sciences, College of Veterinary Medicine, University of Florida, Gainesville, FL, United States

**Keywords:** respiratory sensation, transient inspiratory occlusions, (fMRI ) functional magnetic resonance imaging, neural correlates, cortical and subcortical mapping

## Abstract

Cortical and subcortical mechanosensation of breathing can be measured by short respiratory occlusions. However, the corresponding neural substrates involved in the respiratory sensation elicited by a respiratory mechanical stimulus remained unclear. Therefore, we applied the functional magnetic resonance imaging (fMRI) technique to study cortical activations of respiratory mechanosensation. We hypothesized that thalamus, frontal cortex, somatosensory cortex, and inferior parietal cortex would be significantly activated in response to respiratory mechanical stimuli. We recruited 23 healthy adults to participate in our event-designed fMRI experiment. During the 12-min scan, participants breathed with a specialized face-mask. Single respiratory occlusions of 150 ms were delivered every 2–4 breaths. At least 32 successful occlusions were collected for data analysis. The results showed significant neural activations in the thalamus, supramarginal gyrus, middle frontal gyrus, inferior frontal triangularis, and caudate (AlphaSim corrected *p* < 0.05). In addition, subjective ratings of breathlessness were significantly correlated with the levels of neural activations in bilateral thalamus, right caudate, right supramarginal gyrus, left middle frontal gyrus, left inferior triangularis. Our results demonstrated cortical sources of respiratory sensations elicited by the inspiratory occlusion paradigm in healthy adults were located in the thalamus, supramarginal gyrus, and the middle frontal cortex, inferior frontal triangularis, suggesting subcortical, and cortical neural sources of the respiratory mechanosensation are thalamo-cortical based, especially the connections to the premotor area, middle and ventro-lateral prefrontal cortex, as well as the somatosensory association cortex. Finally, level of neural activation in thalamus is associated with the subjective rating of breathlessness, suggesting respiratory sensory information is gated at the thalamic level.

## Introduction

Respiration at the brainstem level is necessary for maintaining bodily homeostasis, but is usually not sensed at the conscious level during eupneic breathing (Davenport and Vovk, 2009; Chan and Davenport, 2010). Stimulation of respiratory-related afferents results in conscious awareness of respiration. When ventilation is obstructed or challenged, subcortical, and cortical substrates are activated, forming a basis for respiratory interoception including awareness, detection, and discrimination (von Leupoldt and Dahme, 2005; Davenport and Vovk, 2009; Khalsa et al., 2018). Subsequent motor actions or no-actions follow based on cognitive judgments of the sensations. Therefore, accurate respiratory sensation is essential for making appropriate behavioral decisions, such as use of medication. Failure of cognitive awareness in respiratory sensation can lead to life-threatening events (Kifle et al., 1997; Davenport et al., 2000; Davenport and Kifle, 2001).

Cortical mechanosensation of breathing in humans has been measured by recording cortical evoked potentials elicited by short respiratory occlusions, namely, the respiratory-related evoked potential (RREP) (Davenport et al., 1986; Chan and Davenport, 2010; von Leupoldt et al., 2013). The RREP Nf, P1, and N1 peak were identified as the short-latency exogenous components, whereas the P2 and P3 peaks were viewed as the long-latency endogenous components. The short-latency peaks were thought to be modulated by physiological factors such as stimulus intensity and the long-latency ones can be modulated by cognitive or psychological factors such as individuals' affective states (Chan and Davenport, 2010). Cortical sources of the RREP were examined by low- and high-density electroencephalography recording with the Minimum Norm Estimate (MNE) in the past decade (Davenport et al., 1996; Logie et al., 1998; von Leupoldt et al., 2010, 2011). The RREP Source analysis revealed that early cortical signals in response to the single respiratory occlusion paradigm originated from pre-central and post-central dipoles (Logie et al., 1998; von Leupoldt et al., 2010). Other studies further suggested that later signals including N1, P2, and P3 originated from the sensorimotor cortex and right lateral frontal cortex, the midline frontal cortex and sensorimotor cortex, and the parietal cortex, respectively (Davenport et al., 1996; Webster and Colrain, 1998, 2000; von Leupoldt et al., 2010).

The electrophysiological method provides excellent temporal resolution, but lacks spatial details. Neuroimaging methods such as the functional magnetic resonance imaging (fMRI) can have excellent spatial definition but poor temporal associations. Earlier neuroimaging research concerning respiratory sensation generally studied brain substrates activated by chemoreceptor stimulation and mechanoreceptor stimulation (Manning et al., 1992; Banzett et al., 2000; Peiffer et al., 2001, 2008; Moosavi et al., 2003; von Leupoldt et al., 2008, 2009a,b). Specifically, these studies used hypoxia, hypercapnia, breath volume, or resistive loads to induce sensation of dyspnea. It was found that cortical and subcortical substrates associated with these stimuli include amygdala, anterior insula, anterior cingulate cortex, sensorimotor cortex, supplementary motor cortex, and medial thalamus. Other studies used respiratory loads for a few breaths to study interoceptive processing at the cortical level (Peiffer et al., 2008; von Leupoldt et al., 2008, 2009b; Paulus et al., 2012; Stewart et al., 2013; Berk et al., 2015; Haase et al., 2016). Jack et al. (2010) utilized a transient occlusion of inspiration (TIO) method, similar to the occlusion elicited RREP, to study patterns of brain activation in 4 patients with idiopathic hyperventilation (Jack et al., 2010). They found that sensorimotor area, premotor area, and anterior insula were activated in these patients. However, there has been a lack of evidence examining the brain substrates associated with respiratory sensation elicited by transient occlusions in healthy individuals.

The evoked potential studies with respiratory obstructions or loads measure neural processing of respiratory sensory stimuli (Davenport et al., 1986). Previous studies have used this method to examine cortical processing of respiratory sensation in normal adults and children, asthmatic children, patients with lung transplant, and anxious individuals (Davenport et al., 1996, 2000, 2006; Chan et al., 2014, 2015). For example, RREP P1 peak was absent in some life-threatening asthmatic children, indicating a deficient ability in inspiratory load processing at the neural level (Davenport et al., 2000). Chan et al. (2014) found that individuals with generalized anxiety disorder showed a delayed latency for the RREP P3 peak compared to normal controls (Chan et al., 2014). Taken together, the temporal aspects of neural processing of respiratory sensation were found influenced by various physiological and psychological factors. The importance of examining brain substrates with this stimulus lies in the fact that the brain sensory mechanism is essential in understanding neural processing of respiratory sensation (Chan and Davenport, 2010). Therefore, the purpose of this study was to identify cortical and subcortical substrates related to respiratory mechanosensation elicited by the transient inspiratory occlusion. The RREP studies have suggested the cortical sources of the Nf peak are likely located at the frontal cortices, the sources for the P1 peak to be the somatosensory cortices, the sources for the N1 and P3 peaks to be the right lateral sensorimotor cortices and lateral parietal cortices, respectively (Davenport et al., 1996; Logie et al., 1998; Chan and Davenport, 2010; von Leupoldt et al., 2010). Although the RREP studies were unable to provide inferences for subcortical activation patterns in response to respiratory mechanical stimulus, previous animal studies have suggested that stimulation to the phrenic nerve and thalamus resulted in direct inputs to the 3a and 3b areas (Yates et al., 1994; Davenport et al., 2010). In the present study, we collected the functional magnetic resonance imaging (fMRI) data for spatial localizations. We hypothesized that TIO stimuli will elicit neural activation in the thalamus, sensorimotor cortex, frontal cortex, and parietal cortex.

## Methods

### Participants

Twenty-three (13 females) volunteers (mean age = 23.7 ± 3.1 years) participated in this study. The participants were reportedly free of cardio-respiratory diseases and neurological diseases. Women over 50 years old were also excluded for possible menopause-related syndromes. In order to comply with the fMRI scanner requirements, the volunteers were all pre-screened to ensure they were free of metal implants, pacemakers or braces, and claustrophobia. All volunteers signed the informed consent and performed the pulmonary function test before starting the experiment (Miller et al., 2005). Their forced expiratory volume in 1 second (FEV 1) must exceed 70% of the normative values in order to continue the experiment. The study was approved by the institutional review board in the Chang Gung Medical Foundation.

### Respiratory Apparatus

The details of the setting were described previously by Chan et al. (2014). Specifically, the participants breathed with a facemask through a two-way non-rebreathing valve (Hans Rudolph Inc., Kansas City, USA). The inspiratory port of the valve was connected to a customized occlusion valve (Hans Rudolph Inc., Kansas City, USA) which was placed 3 meters away from the scanner. The occlusion valve was connected to pressure tubing to the air tank via a solenoid of a customized trigger outside of the scanner. The experimenter manually controlled the trigger to activate closure of the occlusion valve. The mouth pressure of participants was monitored with a pressure tubing at the center of the non-rebreathing valve connecting to a differential pressure transducer, amplifier (1,110 series, Hans Rudolph Inc., Kansas City, MO, USA) and a PowerLab signal recording unit (ADInstruments Inc., Bella Vista, NSW, Australia).

### Experiment Protocol

After completing the screening procedure, the participant wore a facemask and earplugs, supine in the scanner with the head immobilized before securing the head coil in place. The participants were instructed to breathe normally during the experiment. A short inspiratory occlusion of 150 ms was delivered at the onset of the inspiratory phase every 2–4 breaths. We randomly selected breaths for short inspiratory occlusions across the approximate 12 min of acquisition time, and collected at least 32 successfully occluded breaths. After the experiment, the participants were instructed to rate their level of breathlessness during the experiment based on a Visual Analog Scale (0 = no breathlessness, and 100 = maximal breathlessness).

### Image Acquisition

A T1-weighted image was acquired using a three-dimensional gradient-echo sequence (MP-RAGE) with an isotropic resolution of 1 mm. The fMRI experiment was performed using a 3-T whole-body scanner (Siemens MRI Scanner MAGNETOM Prisma, Erlangen, Germany) with an 8-channel brain array coil. The blood oxygenation level-dependent (BOLD)-based fMRI signal was taken as an indirect measure of local neural activities. At least 32 continuous axial slices (thickness = 3 mm) were acquired by using a gradient echo, echo-planar imaging sequence (TR = 2 s, TE = 30 ms; flip angle = 90 degrees; ASSET = 2, matrix = 64 × 64; field of view = 220 × 220 mm).

### Data Analysis

The fMRI Image processing and statistical analysis was performed with SPM8 (http://www.fil.ion.ucl.ac.uk/spm/, Department of Imaging Neuroscience, University College London, UK) and analysis of functional neuroimages (AFNI) (Cox, 1996). All images were realigned to the first image, spatially normalized into standard anatomical space based on the MNI template and smoothed with an isotropic Gaussian kernel of 6-mm full-width at half-maximum. At the first-level analysis, every short inspiratory occlusion was regarded as an event of RREP that induced corresponding hemodynamic responses, whereas the flat signal fluctuations during normal breathes were taken as the baseline (Birn et al., 2006). The statistical analysis was performed with the general linear model with time and dispersion derivatives, and the six motion parameters were included as nuisance regressors. After the model estimation, the generated beta map for each individual reflected the magnitude of hemodynamic responses induced by transient inspiratory occlusions. At the group level, one-sample group-averaged activation maps were presented at the significant level of AlphaSim corrected *p* < 0.05 (uncorrected *p* < 0.0001, cluster size = 60 voxels). Finally, correlational analyses were conducted to examine the associations between the participants' rating of breathlessness and the averaged beta values within selected regions of interest (ROI), where spherical ROIs were centered at the peak of activated brain areas with radius of 5 mm.

## Results

Table 1 shows the demographics and characteristics of the study participants. There were 13 females and 10 males and the mean (± SD) age of the 23 participants were 23.7 (± 3.1) years. All participants' FEV1 performances exceeded 70% of predicted values and the ratios of functional vital capacity and FEV1 were over 100%. There were on average (±SD) 107 (± 24) non-occluded breaths collected during the 12-min recording time, and the individuals' respiratory frequencies fell in the range of 8–14 breaths/min with an average (±SD) of 11 (±1) breaths/min. The averaged (± SD) rating of breathlessness using the VAS was 20 (± 25. 78) out of 100.

**Table 1:** Demographics and behavioral assessments of participants.

**Variables**	**Mean (*SD*)**
Age (yrs)	23.7 (3.1)
Sex (female/male)	13/10
Forced expiratory volume in 1 s (L)	3.21 (0.47)
Forced expiratory volume in 1 s (% of predicted value)	78 (8.64)
Forced expiratory volume in 1 s/Forced vital capacity (%)	107 (8.83)
Self-reported breathlessness (VAS)	20 (25.78)

**Figure 1:**
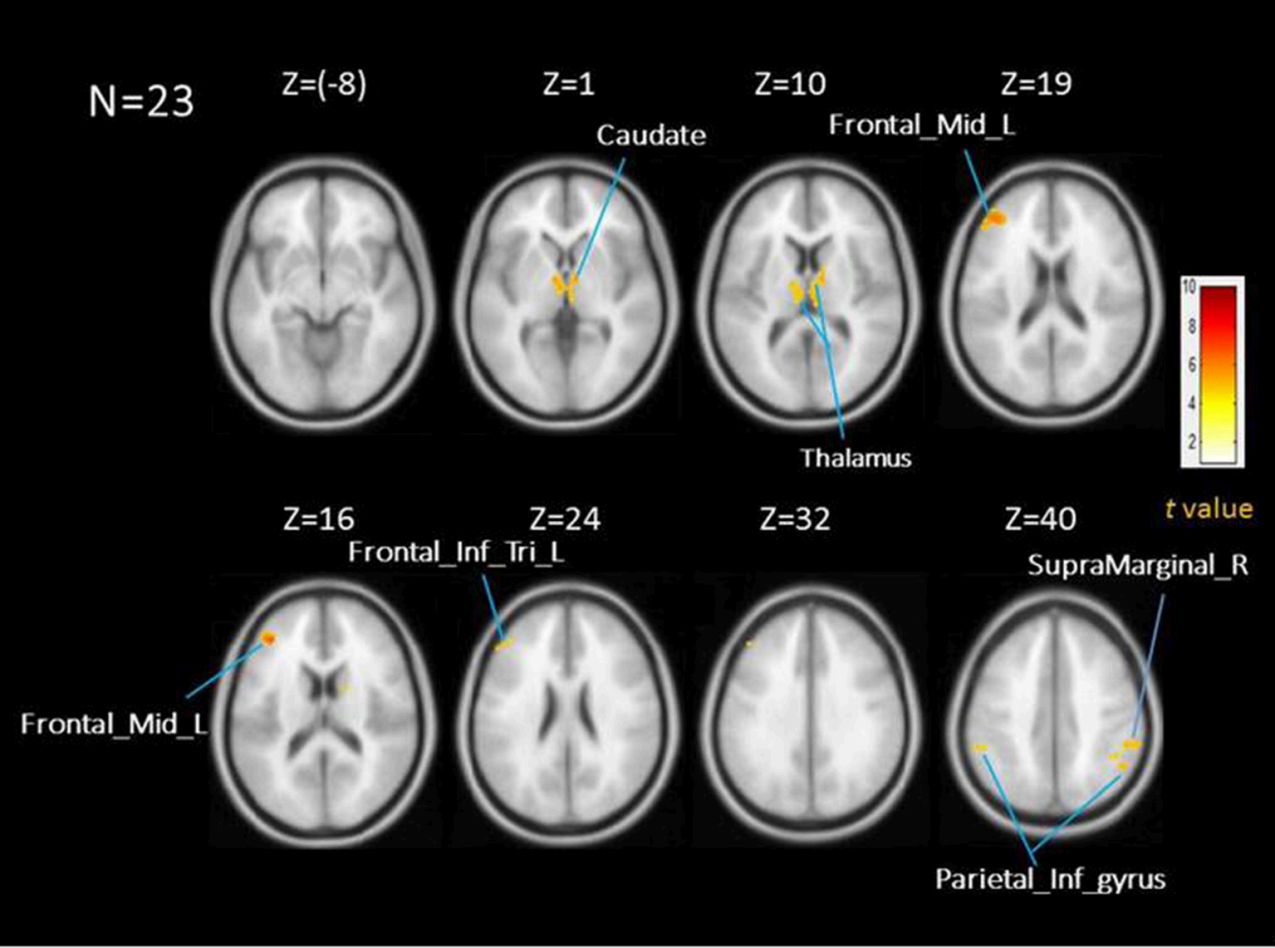
Averaed brain activation map showing the main effect of repsiratory occlusions under AlphaSim corrected *p* < 0.05; time and dispersion derivatives. Areas activated include thalamus, caudate, middle frontal cortex, inferior frontal triangularious, and inferior parietal cortex.

Figure 1 shows the activation map of the effect of inspiratory occlusions for the 23 participants. The significantly activated areas include the right caudate [10, −4, 2], left caudate at extra-neucleus [−8, −4, 2], left thalamus [−2, −14, 4], right inferior parietal gyrus [56, −40, 42], left inferior parietal cortex [−56, −40, 44], left middle frontal cortex [−40, 40, 16], and left inferior frontal triangularis [−50, 36, 22]. The listed coordinates were all in the MNI space and used for the ROI analysis. Table 2 lists the significant clusters activated at the group level during the events of inspiratory occlusions. The main activated brain regions were the right inferior parietal lobe, left middle frontal gyrus, left thalamus, and left inferior parietal lobe (Alpha Sim corrected *p* < 0.05).

**Table 2:** All regions showing significant activations with short inspiratory occlusions; AlphaSim corrected *p* < 0.05 [(uncorrected *p* < 0.0001, cluster size = 60 voxels), T value threshold = 4.589].

**Brain region**	**X, Y, Z**	**(Cluster)**	***T* value**
R Inferior parietal	52,−54,46	194	6.66
R Supra Marginal	56,−40,42		5.65
L Mid Frontal	−40,40,16	201	6.60
L Inferior frontal triangularis	−50,36,22		5.78
L Thalamus	−2,−14,4	395	6.29
R Caudate	10,−4,2		5.95
L Caudate	−8,−4,2		5.51
L Inferior parietal	−56,−40,44	73	5.63

**Figure 2:**
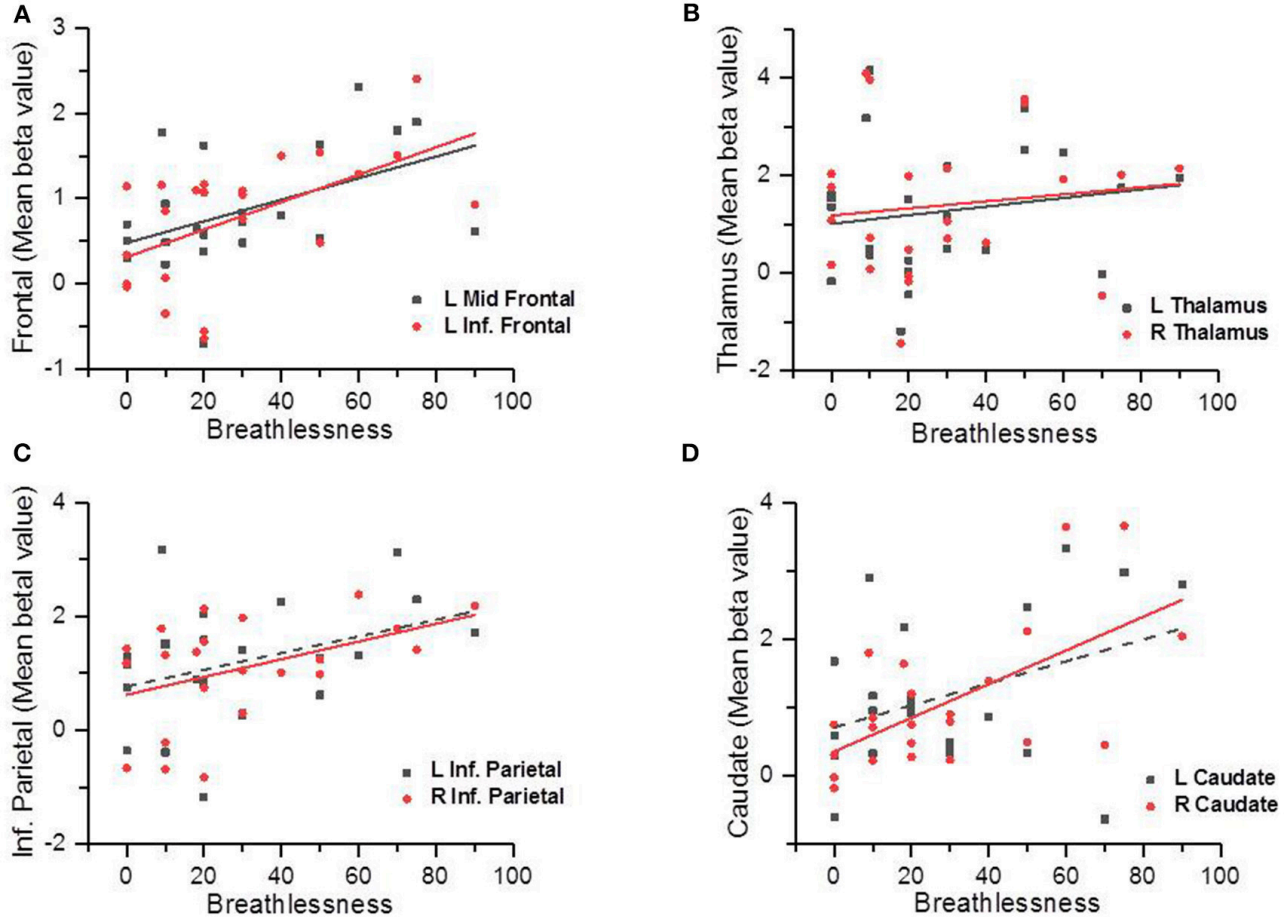
Scatter plots showing the correlations between the participants' subjective ratings on breathlessness and the brain area activation levels (measured by the beta values); the solid line indicated there was a significant correlation (*p* < 0.05) and the dotted line indicated there was no statistically significant correlation (*p* > 0.05). Correlations between self-reported breathlessness level and left middle frontal cortex and left inferior frontal cortex **(A)**, bilateral thalamus **(B)**, bilateral inferior parietal cortices **(C)**, and bilateral caudate **(D)**.

Figure 2 shows the Pearson Correlation Coefficients between the participants' self-rated level of breathlessness (measured by the VAS of breathlessness) and their level of hemodynamic response in each activated area (measured by β values). The behavioral assessments showed the averaged level of breathlessness was significantly correlated with the β values in the bilateral thalamus (L: *r* = 0.61, *p* = 0.002; R: *r* = 0.64, *p* = 0.001), right caudate (*r* = 0.62, *p* = 0.002), right supramarginal gyrus (*r* = 0.6, *p* = 0.002), left middle frontal cortex (*r* = 0.48, *p* = 0.022), and left inferior frontal triangularis (*r* = 0.55, *p* = 0.006). There was no significant correlation between the VAS scores and the beta value of the left inferior parietal cortex (*r* = 0.362, *p* = 0.09), or between the VAS scores and the beta value of the left caudate (*r* = 0.374, *p* = 0.079). Besides, we used the independent *t*-test to compare the differences between brain area activations of the male and of the female subjects, and found no significant difference between these two groups (uncorrected *p* < 0.001, voxel size = 20). Therefore, there was was no gender effect in our results of the present study.

## Discussion

In the present study, we used event-related fMRI analysis to examine the subcortical and cortical neural sources mediating respiratory sensation in response to short inspiratory occlusions in humans. The results indicated that this inspiratory occlusion paradigm indeed induced RREP-based hemodynamic responses among specific brain regions. The occlusion-elicited activation areas include the thalamus, extra-nucleus of caudate, supramarginal gyrus of the inferior parietal lobe, middle frontal lobe, and inferior frontal triangularis.

Our results showed multiple cortical regions activated in response to inspiratory occlusions including the premotor area in the middle frontal gyrus, somatosensory association cortex, but not the postcentral area. We expected to see significant activations in the postcentral area, i.e., the somatosensory cortices, based on previous RREP and source localization reports (Davenport et al., 1996; Logie et al., 1998; von Leupoldt et al., 2010). Logie et al. (1998) used brief inspiratory occlusions to study the RREP with the Electromagnetic Source Estimation program and modeled electrical dipoles for neural generators. They discovered the post-central and pre-central radial dipoles as the locations of the RREP neural generators (Logie et al., 1998). Although in the present study, significant BOLD responses were not observed in these areas, the results were still similar to the previous studies examining the sources of neural activations in response to somatosensory stimulus (Backes et al., 2000; Nihashi et al., 2005; Bak et al., 2011). Bak et al. (2011) found SII area activation during median nerve stimulation, but not the SI cortex. Backes et al. (2000) also found that SI was significantly activated with higher intensity of somatosensory stimulus, but not with a lower level of stimulus. Similarly, with high-density EEG technique, von Leupoldt et al. (2010) reported neural activations at the bilateral frontal and somatosensory cortex during 160-msec respiratory occlusions with small signal magnitudes and unstable sources (von Leupoldt et al., 2010). Post-central gyrus was also not consistently reported as one of the most significantly activated areas in the other studies using respiratory loading techniques (Stewart et al., 2013, 2014; Berk et al., 2015). The inspiratory occlusion in the current study was 150 ms in length and more than 50% of our participants (6 out of the 10 males and 7 out of the 13 females) rated the difficulty in breathing lower than 21 out of 100 in the rating scale. This low level of respiratory mechanostimulation may explain why the somatosensory cortex was not identified as a significantly activated area in our study.

The premotor area in the middle frontal lobe and the left inferior frontal triangularis in the vetro-lateral prefrontal gyrus were activated with this occlusion paradigm. Previous RREP results have suggested the premotor area as possible neural sources for the Nf peak (Chou and Davenport, 2007; Chan and Davenport, 2010). In addition, von Leupoldt et al. (2010) found neural sources at the lateral sensorimotor cortices and the right lateral frontal cortex for N1 peak where as our results suggested the sources may be at the ventro-lateral prefrontal cortex. Loading studies conducted by Paulus et al. (2012) and Stewart et al. (2013) also reported that middle frontal gyrus, inferior frontal gyrus in addition to inferior parietal lobe and thalamus were significantly activated during loaded breathing (Paulus et al., 2012; Stewart et al., 2013). Binks et al. (2014) investigated the time course of cortico-limbic neural responses to air hunger and found that with the on-transition and steady state air hunger conditions, BOLD signals changed in the premotor area, and middle frontal gyrus which supported our results in the current study (Binks et al., 2014). The experimental paradigm in their study also elicited increased activations in the insula and dorsal anterior cingulate cortex, which are usually associated with aversive response to the volume-controlled ventilation. Our respiratory transient obstruction was not associated with long period of loading or restricted volume, and therefore was reasoned to elicit limited activation in the limbic systems in normal healthy subjects.

Significant neural activation was observed in the supramarginal gyrus in bilateral inferior parietal cortices, i.e., the SII area. This result is consistent with some previous reports of the fMRI studies with somatosensory stimulus and respiratory loading techniques (Ferretti et al., 2004; Chen et al., 2008; Bak et al., 2011; Stewart et al., 2014). The SII area is known to receive sensory information from the S1 cortex and is associated with interpreting sensory stimuli and interacting with attention (Chen et al., 2010; Stewart et al., 2014). Based on previous RREP reports, the sources of longer-latency peaks such as P2 and P3, are difficult to localize and were suggested to have origins in the somatosensory association cortex, although one earlier report of von Leupoldt, et al.'s (2010) found the dipole source of P3 in the lateral instead of inferior parietal cortex (von Leupoldt et al., 2010).

Our correlational analyses revealed that right thalamus activation is the most highly associated with the participants' reported level of breathlessness, and to a lesser extent the right caudate, left thalamus, and activated cortical structures. Galli et al. (2013) reported that left caudate activation was significantly associated with the self-rated VAS unpleasantness in breathlessness in men and with task reaction time in women (Galli et al., 2013). Taken together, the above evidence suggests that the thalamus and right caudate are most likely mediating the sensory component of breathlessness, whereas the left caudate may mediate the affective component of breathlessness. In addition, among the significant correlated areas, subcortical and cortical structures in the right hemisphere seem more associated with breathlessness than the left, suggesting possible laterality in respiratory sensory processing. The cause of this laterality is unclear, but the result in the present study seems consistent with the previous studies in RREP (Chou and Davenport, 2007; Davenport et al., 2007), and preliminary data in respiratory sensory processing using Magnetoencephalography in our laboratory (unpublished data).

The results of the present study suggested that brain activation areas in response to short inspiratory occlusions in healthy individuals involved subcortical and cortical areas such as the thalamus, caudate, parietal cortex, and frontal cortex. Compared to some previous studies in dyspnea in normal controls or clinical populations, brain areas related to emotional processing such as the anterior insula, cingulate gyrus, and amygdala were not found activated in this study (Peiffer et al., 2008; Jack et al., 2010; Binks et al., 2014). It is possible that with our current experimental paradigm, the emotion-related brain areas are not activated prominently in normal healthy population. It will interesting, however, to test this implication in individuals with different emotional states, as our results in this study provided a basis for future examinations on influences of other biological and psychosocial factors on brain activation areas elicited by respiratory stimuli.

One limitation of the current study is that our study design could not have ruled out the possibility of including the neural activations in the cortical and subcortical areas associated with the following breath after immediately after the occlusion. Respiratory sensory stimulus may elicit patterns similar to short-term potentiation in respiratory output (Mifflin, 1997). Mifflin (1997) studied rats with high-frequency electrical stimulation of the carotid sinus nerve (CSN) and found that brief CSN stimulation can modulate NTS neuron activity. However, the stimulation was performed in anesthetized rats and therefore cortical and/or subcortical response were not observed with the prep. Future investigation is encouraged to further clarify between these activations from different sources. Another limitation was that other physiological activations such as the participants' heat beats were not accounted as a nuisance regressor in the first level analysis. This was due to the hardware failure of the physiological monitor unit in the current scanner (PRISMA, Siemens) after 5-min signal recording. Future investigations should improve the analysis with the regressor of other possible physiological activations if possible. Future studies are also encouraged to clarify the activation patterns among different sub-groups such as people with higher and lower perceptions of breathlessness, and the impact of emotions on brain activation patterns in respiratory sensation.

## Ethics Statement

This study was carried out in accordance with the recommendations of the guidelines in the Institutional Review Board with the written informed consent from all subjects. All subjects gave written informed consent in accordance with the Declaration of Helsinki. The protocol was approved by the Institutional Review Board of Chang Gung Medical Foundation.

## Author Contributions

P-YC and C-HC conceived and designed the experiment. Y-TW performed the experiment and assisted with creating figures and tables. P-YC and CW analyzed and interpreted the data. P-YC and CW drafted and finalized the manuscript. H-LL, F-ZS, C-YL, and PD assisted with conceptualization of the experiment as well as critical review and editing of the manuscript. All authors contributed to manuscript revision, read, and approved the submitted version.

### Conflict of Interest Statement

The authors declare that the research was conducted in the absence of any commercial or financial relationships that could be construed as a potential conflict of interest.
